# UV-Writing of a Superstructure Waveguide Bragg Grating in a Planar Polymer Substrate

**DOI:** 10.3390/s17091964

**Published:** 2017-08-25

**Authors:** Manuel Rosenberger, Bernhard Schmauss, Ralf Hellmann

**Affiliations:** 1 Applied Laser and Photonics Group, University of Applied Sciences Aschaffenburg, 63743 Aschaffenburg, Germany; ralf.hellmann@h-ab.de; 2 Institute of Microwaves and Photonics, University of Erlangen-Nuremberg, 91054 Erlangen, Germany; bernhard.schmauss@fau.de

**Keywords:** planar Bragg grating, polymer, phase mask, integrated waveguide, excimer laser

## Abstract

We report on the fabrication of a superstructure Bragg grating in a planar polymer substrate. Based on a twofold illumination process an integrated waveguide and a superstructure Bragg grating are subsequently written into bulk polymethylmethacrylate by UV-induced refractive index modification. The measured reflected spectrum of the superstructure Bragg grating exhibits multiple reflection peaks and is in good agreement with performed standard simulations based on the beam propagation method and coupled mode theory algorithms. By applying a varying tensile load we determine the strain sensitivity to be about 1.10 pm/µε and demonstrate the applicability of the superstructure Bragg grating for strain measurements with redundant sensing signals.

## 1. Introduction

Bragg gratings are wavelength-selective filters that are based on a periodical refractive index perturbation inside an optical waveguide. This refractive index perturbation with a period *Λ* results in the reflection of a small wavelength band around the Bragg wavelength *λ_B_* which is given by *λ_B_* = *2n_eff_Λ* with *n_eff_* being the effective refractive index. While Bragg gratings with a single reflection peak are mainly applied, Bragg grating structures with multiple reflection peaks can be easily fabricated as well. For example, superimposed Bragg gratings have been reported to be written in pre-strained germanium doped silica fibers using only a single phase mask [1]. Othonos et al. described the fabrication of seven superimposed gratings in hydrogen-loaded silica fibers while Arigiris et al. studied the effects of superimposing ten gratings in a hydrogenated boron co-doped fiber [2,3]. Such superimposed grating structures have already been used for a tunable fiber laser or for multi-parameter sensing [4,5]. However, a more effective approach to obtain a reflected spectrum with multiple reflection peaks is the fabrication of superstructure Bragg gratings. These devices have successfully been created in germanosilicate planar waveguides and applied for WDM applications [6,7]. Furthermore, Sparrow et al. reported on integrated superstructure gratings in planar silica on silicon substrates [8]. So far, all these devices are based on silica, while polymer materials are rarely used. Only Binfeng et al. recently described the fabrication of superstructure Bragg gratings in a polymer photoresist [9]. However, those structures were fabricated on a brittle and rigid silicon wafer, hampering its applications e.g., in deformation sensing. Since polymer materials exhibit some favorable advantages, e.g., a low Young’s modulus, a high breaking elongation, photosensitivity and biocompatibility, polymer-based structures might find various applications [10,11].

In earlier studies, we already demonstrated that polymer planar Bragg gratings (PPBG) which are written using a single writing step can be applied for different sensing applications like measuring temperature, relative humidity, or tensile and compressive strain [12,13,14,15]. A further development of the PPBGs by fabricating advanced grating structures exhibits great potential for redundant sensors and multi-parameter sensing. In this letter, we focus on the simulation and fabrication of superstructure Bragg gratings in bulk polymer substrates. We describe the spectral characteristics of such multi-reflective devices and discuss the influence of the dimensions of the superstructure based on performed simulations and written Bragg gratings. In addition, we demonstrate the applicability of the polymer planar superstructure Bragg gratings in strain sensing.

## 2. Simulation and Fabrication

### 2.1. Simulation

**Figure 1:**
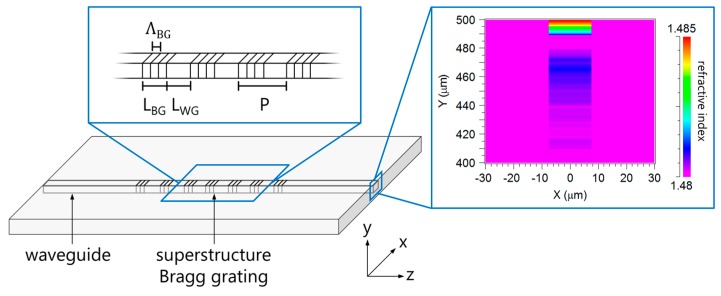
Layout of the superstructure Bragg grating in planar PMMA. The right inset depicts the in-depth refractive index profile, i.e., the index variation in *y*-direction.

The schematic illustration of the superstructure Bragg grating in a planar polymer substrate is illustrated in Figure 1. The superstructure Bragg grating consists of seven Bragg grating segments that exhibit the grating period *Λ_BG_* and are separated by unperturbed regions. The length of a Bragg grating segment is denoted as *L_BG_* and the length of an interrupting region as *L_WG_*. Their sum represents the period of the superstructure *P* = *L_BG_ + L_WG_*. The width of the integrated waveguide is 15 µm whereas the height of the waveguide is based on a refractive index depth profile induced by the UV-writing process which has already been described in Reference [16]. This refractive profile of the integrated waveguide is depicted in Figure 1 revealing a refractive increase of 4.0 × 10^−3^ at the surface of the polymer substrate.

**Figure 2:**
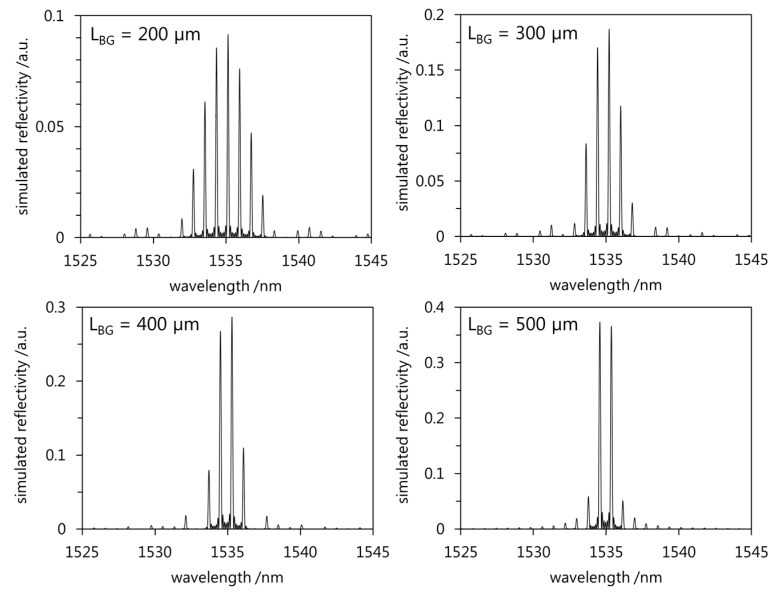
Simulated reflected spectra of superstructure Bragg gratings with the same superstructure period (*P* = 1 mm) and different *L_BG_*.

The spectral characteristics of the superstructure gratings comprise the number of peaks located within the 3 dB range with respect to the peak wavelength which is denoted as *N_3dB_* and the spectral intervals between two neighboring reflection peaks Δ*λ_s_*. *N_3dB_* be calculated according to

(1) $$N_{3dB}=\frac{P}{L_{BG}},$$

while Δ*λ_s_* is given by:

(2) $$\Delta \lambda_{S}=\frac{{\lambda_{B}}^{2}}{\left(2n_{g}P\right)},$$

with *n_g_* being the group index of the guided mode (*n_g_* ≈ *n_eff_*) [9,17,18]. The layout of the polymer planar superstructure waveguide Bragg grating (depicted in Figure 1) is designed using RSoftCAD (Synopsys Inc., Mountain View, CA, USA). Beyond standard geometrical and refractive index layout, for our simulations we included the real in-depth refractive index profile as being measured by sophisticated phase shifting Mach-Zehnder-Interferometric approach as described in [16]. Following, BeamPROP (Synopsys Inc., Mountain View, CA, USA), which is based on beam propagation method, is applied for the simulation of the guided mode. The simulated mode is subsequently employed for the calculation of the reflected spectra by coupled mode theory algorithms using GratingMOD (Synopsys Inc., Mountain View, CA, USA). Figure 2 illustrates the simulation results by showing the reflected spectra of superstructure Bragg gratings. During the simulations *P* is set to be 1 mm while the length of the grating segments *L_BG_* is changed and amounts to 200 µm, 300 µm, 400 µm, and 500 µm. The Bragg grating period is selected according to the available phase mask which exhibits a period *Λ_PM_* of 1036.79 nm. Since the resulting Bragg grating period is given by *Λ_BG_* = *Λ_PM_/*2 the grating period is 518.395 nm.

The simulations show that the number of peaks in the 3 dB range changes according to Equation (1) while the spectral intervals between two neighboring reflection peaks Δ*λ_s_* yields around 0.80 nm and remains constant for different *L_BG_*.

### 2.2. Fabrication Process

Bulk polymethylmethacrylate with a thickness of 1.1 mm was used as a substrate material for the Bragg grating structures. The polymer platelets are cut to size with a mechanical precision saw (Secotom-15, Struers GmbH, Willich, Germany) and the end faces polished with an advanced preparation system (Tegramin-20, Struers GmbH, Willich, Germany) generating a good surface quality suitable for optical fiber coupling.

**Figure 3:**
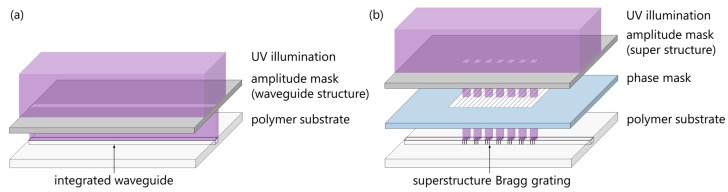
Schematic illustration of the setup (vertically exploded assembly drawing) for the fabrication of superstructure Bragg gratings by successively writing (**a**) an integrated waveguide and (**b**) a superstructure Bragg grating (masks and polymer substrate are in contact).

The superstructure waveguide Bragg grating is realized in a twofold illumination process. While Wochnowski et al. applied a twofold UV-based fabrication process to fabricate polymer planar waveguide Bragg gratings, superstructure Bragg gratings have not been reported to be written in planar polymer substrates [19]. Initially, an integrated waveguide is inscribed into the polymer substrate. Therefore, the polymer substrate is covered by an amplitude mask that exhibits a 15 µm wide waveguide structure and is subsequently illuminated using a KrF excimer laser emitting a wavelength of 248 nm (Figure 3a). The waveguide is written with a pulse frequency of 200 Hz, a pulse fluence of 8 mJ/cm^2^, and a UV-dosage of 24 J/cm^2^. As already mentioned the UV illumination induces a distinct refractive index depth modification resulting in an integrated waveguide structure as reported in [16]. Secondly, a phase mask with a grating period of 1036.79 nm is placed into contact with the polymer waveguide and subsequently covered by an amplitude mask exhibiting a superstructure (Figure 3b). The superstructure of the amplitude mask exhibits a sample period *P* of 1 mm whereas a length of 500 µm is transparent and applied to create the Bragg grating segments. The superstructure Bragg grating consists of seven grating segments and is written with the identical parameters as the integrated waveguide. After UV-illumination the surface of the polymer substrate is investigated using confocal laser scanning microscopy.

## 3. Results and Discussion

**Figure 4:**
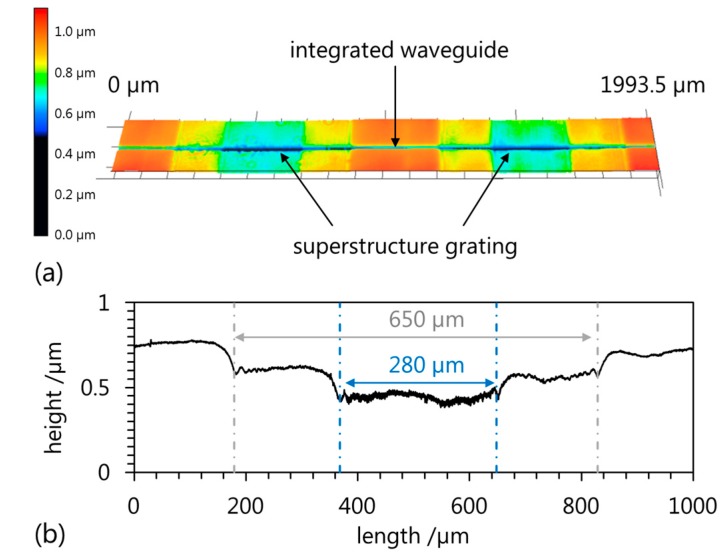
(**a**) Confocal laser scanning microscopy of the surface of a superstructure polymer planar Bragg grating indicating two grating segments and (**b**) height profile of one grating segment.

Figure 4a shows the integrated waveguide and two grating segments of the superstructure Bragg grating. The profile of one grating segment is depicted in Figure 4b and indicates two regions that are differently influenced by the UV-illumination. A 650 µm long region which is slightly affected exhibits a depth of around 165 nm and contains a deeper region with a length of 280 µm and an overall depth of around 300 nm.

This, however, does not match the design of the amplitude mask which would imply a 500 µm long grating segment at the polymer surface. This discrepancy can be attributed to the specific stacking arrangement of the different masks used in the fabrication setup for the generation of the superstructure grating. Based on the arrangement shown in Figure 3b, the fabrication of the superstructure grating can be compared to a proximity illumination causing diffraction behind the amplitude mask. Since only the part of the grating which is not affected by the diffraction contributes to the reflection, the length of the Bragg grating segments *L_BG_* can be considered to be 280 µm.

**Figure 5:**
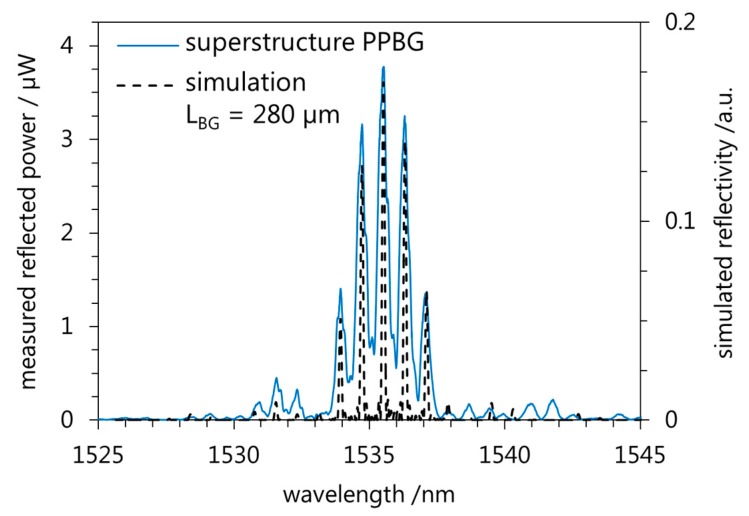
Measured reflected spectrum of the fabricated superstructure PPBG in comparison with a simulation based on *L_BG_* being 280 µm.

For the optical characterization of the superstructure Bragg grating a single mode fiber is connected to the integrated waveguide applying UV-curable glue. Furthermore, a Bragg interrogator consisting of a tunable laser diode, an optical circulator, and a photodiode is used to record the reflected spectrum. Figure 5 shows the measured reflected spectrum of the superstructure Bragg grating in comparison to the simulation with *L_BG_* being 280 µm (*P* = 1 mm). Technically, the measured spectrum has a spectral offset of 0.64 nm as compared to the simulation. However, since polymer Bragg gratings are sensitive to changes in temperature and/or relative humidity, this offset could have been caused by the laboratory conditions [20]. Hence, for a better comparability we readjusted the center peak wavelength of the simulated spectrum to correspond to the center peak wavelength of the measured spectrum.

The measured spectrum exhibits a center peak wavelength at 1535.52 nm while the two neighboring wavelength peaks amount to *λ_B_*_(−1)_ = 1534.73 nm and *λ_B_*_(+1)_ = 1536.30 nm resulting in spectral intervals Δ*λ_s_* of 0.79 nm and 0.78 nm. Following (2) Δ*λ_s_* calculates to be 0.80 nm which demonstrates the good agreement of the measured values of the spectrum to the simulation results. Furthermore, the reflected spectrum of the superstructure Bragg grating shows three reflection peaks (*λ_B_* and *λ_B_*_(*±*1)_) which are located within the 3 dB range. According to (1) this strengthens the assumption that *L_BG_* is in the range of 280 µm In addition, the results show that the simulation based on the Bragg grating segment *L_BG_* being 280 µm is in excellent agreement with the measured superstructure Bragg grating. This in turn allows the design of prospective amplitude masks for the fabrication of superstructure Bragg gratings with specific spectral characteristics. Overall, the proposed UV-based fabrication technique allows the writing of superstructure waveguide Bragg gratings directly into a polymer substrate establishing numerous possible applications in wavelength division multiplexing and optical sensing.

**Figure 6:**
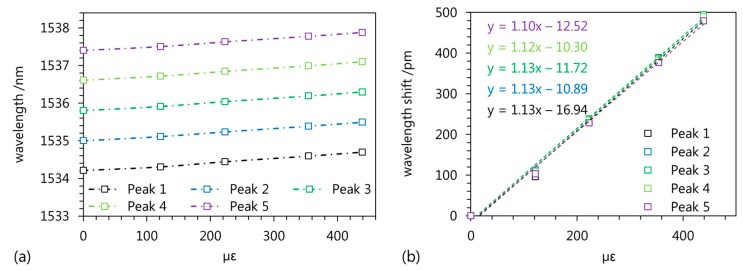
(**a**) Spectral position and (**b**) relative wavelength shift of the five reflected wavelength peaks of the superstructure PPBG during tensile strain.

To highlight the applicability of superstructure Bragg gratings, their strain sensitivity has been investigated. Therefore, a tensile testing machine is used to load the polymer chip with tensile strain while the spectral positions of five reflected wavelength peaks are recorded. Figure 6a shows the spectral position of the reflected wavelength peaks over the applied strain revealing a linear and comparable behavior of the five sensor signals. The sensitivity of the reflected wavelength is in the range of 1.10 pm/µε to 1.13 pm/µε (Figure 6b) which is close to the sensitivity of Bragg grating sensors based on polymer optical fibers [21]. Hence, the experimental results reveal that the described fabrication process allows the writing of superstructure polymer planar waveguide Bragg gratings which can be used for strain measurements with redundant sensing signals. 

## 4. Conclusions

We have described a UV-writing process for the fabrication of superstructure planar waveguide Bragg gratings in polymer substrates and show that the resulting spectral characteristics are in excellent agreement with simulation results, which allows for an engineering of multi-reflective sensing devices. In our particular structure, the superstructure Bragg grating exhibits multiple reflection peaks of which three are located in the 3 dB range separated by spectral intervals of 0.79 nm and 0.78 nm. Furthermore, we show that the superstructure PPBG exhibits a linear strain sensitivity in the range of 1.10 pm/µε to 1.13 pm/µε making the device applicable for tensile strain sensing with redundant sensor signals.
